# Response: Commentary: Facial Width-to-Height Ratio (fWHR) Is Not Associated with Adolescent Testosterone Levels

**DOI:** 10.3389/fpsyg.2018.00160

**Published:** 2018-02-19

**Authors:** Carolyn R. Hodges-Simeon, George B. Richardson, Katherine N. Hanson Sobraske, Theodore Samore, Michael Gurven, Steven J. C. Gaulin

**Affiliations:** ^1^Department of Anthropology, Boston University, Boston, MA, United States; ^2^School of Human Services, University of Cincinnati, Cincinnati, OH, United States; ^3^Department of Anthropology, University of California, Santa Barbara, Santa Barbara, CA, United States; ^4^Department of Anthropology, University of California, Los Angeles, Los Angeles, CA, United States

**Keywords:** facial width-to-height ratio, testosterone, puberty, adolescence, sex hormones, facial dimorphism, secondary sexual characteristics

We thank Welker et al. (2016) for their interesting commentary and helpful additional analysis of our recent article. Given continuing interest in the relationships between facial width-to-height ratio (fWHR) and behaviors such as aggression and dominance (e.g., Carré and McCormick, 2008; Ozener, 2012; Lefevre et al., 2014; Geniole et al., 2015), it is important to address the potential role of pubertal testosterone (T) in facial development.

Hodges-Simeon et al. (2016) set out to evaluate the theory that fWHR is a sexually selected signal of T and T-derived traits. According to this theory, fWHR provides unique information about T that cannot be easily inferred from more obvious parameters such as body size, thereby facilitating predictions about aggression, strength, and dominance (Weston et al., 2007). To this end, Hodges-Simeon et al. (2016) conducted five tests based on the ontogenetic pattern exhibited by secondary sexual characteristics: (1) evidence of trait growth that is temporally contiguous with the beginning of mating competition (i.e., puberty in humans); (2) evidence of a growth spurt in the trait parallel to the growth spurt in T; (3) association with other known T-dependent traits; (4) association with T; (5) association with T after controlling for age (to target developmental associations specifically) and other relevant confounds (i.e., adiposity or BMI; Geniole et al., 2015). Other known T-dependent secondary sexual characteristics show all of these features. For instance, in this sample, vocal fundamental frequency (i.e., pitch) strongly correlates with age (*r* = −0.78, *p* < 0.001), shows a clear growth spurt (Hodges-Simeon et al., 2013), closely correlates with strength (*r* = −0.84, *p* < 0.001) and T (*r* = −0.75, *p* < 0.001), and is associated with T when age is controlled (*r* = −0.38, *p* < 0.01; Hodges-Simeon et al., 2015). fWHR failed all but one of these tests. Thus, Hodges-Simeon et al. (2016) concluded that their findings “add to doubts about the status of fWHR as a sexually-selected signal for pubertal T and T-derived traits” (p. 12).

Both Hodges-Simeon et al. (2016) and Welker et al. (2016) found that fWHR was significantly associated with T when age was controlled. Hodges-Simeon et al. (2016) addressed this relationship in their discussion, pointing to a potential residual effect of prenatal T as a possible explanation (however, see Whitehouse et al., 2015). Below, we devote additional substantive and analytic attention to the structure connecting fWHR, T, and age, as well as the potential usefulness of inferring attributes such as strength from fWHR. We then discuss the Welker et al. (2016) criticism of the Hodges-Simeon et al. (2016) age range and also their comment about variable transformation. Finally, we discuss future directions for research examining the potential effect of T on fWHR.

## fWHR and age

**Figure 1:**
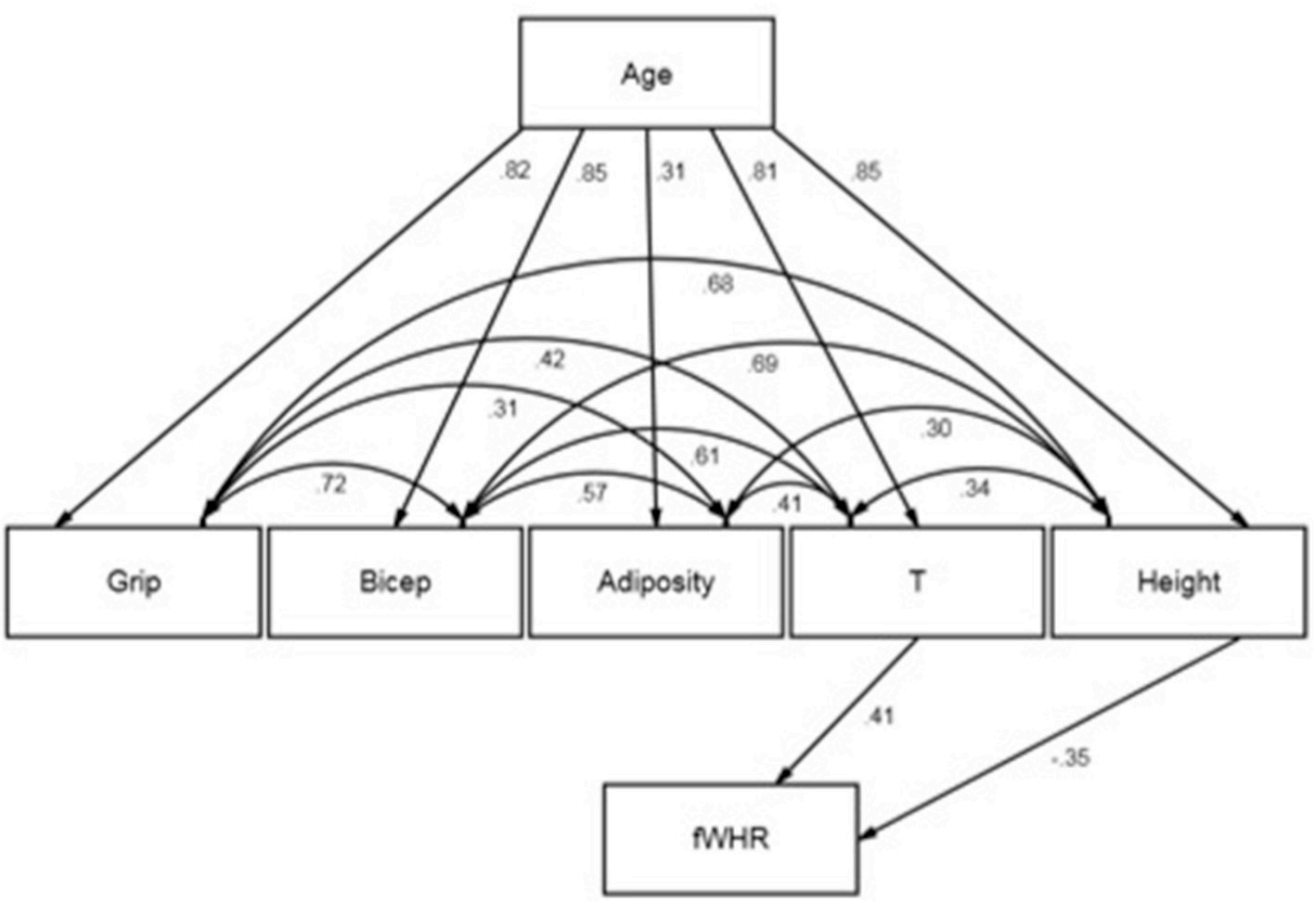
Structural equation model note. Standardized estimates displayed. Effect of adiposity on fWHR omitted due to non-significance.

In their multiple regression, Hodges-Simeon et al. (2016) found a moderate negative effect of age on fWHR and a moderate positive effect of T on fWHR. We examined our estimates again and observed that age did not have a significant bivariate association with fWHR. This is unusual given that a variable, Z (age in this case), which reverses or changes an association between X and Y (T and fWHR in this case) when conditioned upon, typically has bivariate associations with both X and Y (Pearl, 2014). We also noted that many of the variables under study were highly correlated (e.g., T and height and age). This makes substantive sense because these variables are strongly age-scheduled. Taken together, our observations led us to conclude that multicollinearity could have plagued our fifth test as well as the Welker et al. (2016) analyses. We found that this was likely given that *R*^2^ was substantial yet no *p*-values were smaller than 0.10, and given that variance inflation factors were observed at 4.2 for age, 3.3 for T, and 4 for height. Thus, we carried out a secondary analysis of our data using structural equation modeling (SEM; see Figure 1). SEM enabled us to model the structure connecting our predictor variables as well as determine whether fWHR should be regressed directly on age.

## SEM

Given that age was uncorrelated with fWHR and seemed plausibly exogenous to the other variables, we reasoned that T, height, and adiposity explained age-scheduled changes in fWHR. That is, we theorized that age and fWHR might be statistically independent conditional on these variables. We also included handgrip strength and bicep size in our model so that we could evaluate how much unique information fWHR provided about these variables. Age was exogenous in the model while T, height, adiposity, handgrip strength, and bicep size covaried and reflected age. fWHR reflected T, height, and adiposity. We used robust maximum likelihood (MLR) as the estimator and full information maximum likelihood (FIML) to handle missing data. We tested our hypothesized model and fit to the data was excellent (*X*^2^ = 1.18 (2), *p* = 0.55; CFI = 1.00; RMSEA = 0.00 [0.00–0.18]). This indicated that constraining the direct effects of age, handgrip strength, and bicep size to zero did not introduce significant misfit. Moreover, the effect of adiposity was non-significant. Importantly, the effects of height (–) and T (+) were moderate and opposite in sign. The *indirect* effects of age on fWHR, through T and height, were roughly moderate in magnitude, opposite in sign, and significant at *p* < 0.05. The correlations between T, adiposity, and height, not accounted for by age, were moderate to large in magnitude (see Figure 1).

Our results suggest that age is indeed associated with fWHR, but only within levels of T and height. Among those with the same T level, older participants have lower fWHR and this is explained by their greater heights. For those with the same height, older participants had greater fWHR and their higher T levels explain this. This means that in the bivariate case, age and fWHR are uncorrelated because as age increases, it has effects through height and fWHR that cancel one another out (i.e., statistical suppression is occurring). To reproduce our original findings and those of Welker et al. (2016), we regressed fWHR directly onto age and this model produced an estimate of negative sign, similar to that reported by Hodges-Simeon et al. (2016). The advantage of our SEM analysis is that a test of model fit to the data was available to evaluate whether that age effect on fWHR was nil. Our findings suggest that fWHR reflects age indirectly and should not be regressed directly upon it.

To assess the unique information that fWHR might provide about T-derived traits not accounted for by height and adiposity, which are easily detected, we estimated the indirect associations of fWHR with grip strength and bicep size. We found that *holding age constant*, fWHR provided significant indirect information about handgrip strength via height but not T, and significant indirect info about bicep size via T and also height. It seems, then, that fWHR provided some unique information about bicep size that could not be inferred from height (β = 0.27 or about 7% of variance), whereas height provided information about handgrip strength and fWHR did not. Taken together, these findings suggest that fWHR could have been useful for sizing up same-age competitors. However, this would require that pubertal males were either (a) divided into groups homogeneous on age or (b) able to condition their judgements of potential dominance or aggression on age. As noted by Hodges-Simeon et al. (2016):

Furthermore, arguments about the signal value of fWHR must consider the ecological context and hence validity of the alleged message. What does it mean to say that the message only has content when the observer “controls for age”? Many developmental traits loosely co-vary with age and it is these traits, rather than age, that would have been the basis of inter-individual judgments. The signaler's age is unlikely to have been an independent variable that observers could have used to adjust their perceptions in ancestral populations (p. 11).

## Age range

Welker et al. (2016)'s primary point of criticism is that Hodges-Simeon et al. (2016) use an overly liberal age range in their study design. The sample in question ranges from 8 to 22—a period of enormous phenotypic change in males. Any secondary sexual characteristic—i.e., those traits under the ultimate influence of sexual selection and often the proximate influence of androgens like T—shows strong, observable changes during this time. Therefore, the observation that both T and T-dependent traits are more masculinized in a 20-year-old male compared to his 10-year-old self is a banal fact. Barring a rare pathology, a 20-year-old will have a substantially lower voice, taller height, and greater T than he did when he was 10. Because of this, any cross-sectional dataset that includes 10–20 year olds will show a strong association between age and any T-dependent secondary sexual characteristics. Therefore, any purported secondary sexual characteristics should show evidence of enhanced growth during adolescence, operationalized as a significant zero-order correlation within a sample with this kind of age range. Indeed—as Welker et al. (2016) point out—most phenotypic change usually happens between the ages of 12–16 in samples from energy-abundant countries. As the authors concede, an age range of 12–18 would be more appropriate for the Tsimane (where development is slower than in the US and growth is stunted), yet an expanded age range did not yield significant results. While we agree that a narrower age range may have improved the study in other ways and that the titular use of “adolescent” may create slightly different expectations, the liberal age range (and larger N) used should make the hypothesized effect of T on fWHR easier to detect rather than more difficult. For instance, the correlation between age and T is stronger for the entire sample (*r* = 0.83, *p* < 0.001) than for the limited, 12–16 sample (*r* = 0.57, *p* < 0.001).

## Variable transformation

As a final point, Welker et al. (2016) contended that T was only transformed for the multiple regression model, but not the zero-order correlations. This was not the case. We see that the authors might have been confused by the description of our data analysis (i.e., “For regression analyses, T, height, strength, voice pitch, and age were log-transformed to match Pearson's correlation assumption of normality.”); however, the same transformed variables were used to generate both zero-order and multivariate correlations.

## Discussion

Using SEM, we found (a) opposite-valence indirect effects of age on fWHR through height (−) and T (+) indicating that the absence of a significant bivariate correlation between age and fWHR was attributable to statistical suppression; (b) conditional independence of age and fWHR, which implied that the latter should not be regressed directly upon the former; and (c) fWHR did not provide much useful information about T or T-derived traits, consistent with the Hodges-Simeon et al. (2016) conclusion that “results add to doubts about the status of fWHR as a sexually-selected signal for pubertal T and T-derived traits” (p. 12).

While our findings are consistent with a direct effect of T on fWHR, hitherto no theoretical rationale has been advanced to identify the mechanisms propagating this effect from the former to the latter. While the literature provides a proximate understanding of the pathway through which T affects fundamental frequency via androgen receptors on the vocal folds (Voelter et al., 2008), we have no similar leads on how T might affect fWHR. Moreover, adult fWHR is not associated with either prenatal (Whitehouse et al., 2015) or adult testosterone (Bird et al., 2016). Finally, it's not clear that fWHR is sexually dimorphic (Kramer et al., 2012; Lefevre et al., 2012; Ozener, 2012; Kramer, 2017; however, see Weston et al., 2007; Geniole et al., 2015). Clearly, the unstable relationship with sex is problematic for the claim that fWHR is influenced by pubertal T.

There are also as many as seven alternative hypotheses that have been presented to explain the fWHR association with T (see research and discussion by Haselhuhn et al., 2013; Hehman et al., 2015; Whitehouse et al., 2015; Zebrowitz et al., 2015; Eisenbruch et al., 2017; Kramer, 2017). One hypothesis that has been under-examined thus far is that the fWHR-behavior relationships are a byproduct of the association between fWHR and other relevant traits. Because faces comprise a complex suite of intercorrelated traits, and humans are highly sensitive to even very small variations in facial dimensions, any study of fWHR must be mindful of whether and how much this singular dimension interacts with other aspects of craniofacial masculinity (Dixson, 2017). Future research on fWHR should identify (1) whether certain components of fWHR (e.g., nose length vs. philtrum length vs. eyebrow height) contributes more to social perceptions; and (2) the degree to which fWHR correlates with other sexually dimorphic, T-dependent facial traits (e.g., mandibular length and breadth). Multilevel selection experiments could be useful for this goal (see Brooks et al., 2015).

## Conclusion

We appreciate Welker et al. (2016)'s attention to our study and the topic of potential hormonal influences on adolescent facial growth. The research on fWHR thus far has been intriguing—in particular, findings on the associations between low fWHR and rated or actual aggression, dominance, strength (e.g., Carré and McCormick, 2008; Ozener, 2012; Lefevre et al., 2014; Geniole et al., 2015). Taken as a whole, the surfeit of converging evidence casts doubt on the hypothesis that fWHR is a sexually selected signal of T and T-derived traits. However, T may well play a causal role in the development of greater fWHR. It is important to note that the latter does not necessarily imply the former. Future research should hold height but not age constant if regression is used to test the effect of T on fWHR in other samples with this age range. Structural equations can be used to determine how much unique information fWHR provides about T-derived traits via T. However, because humans were not disaggregated into age groups during evolution, the bivariate association between T and fWHR (without controlling for age) may better inform evaluations of the role of fWHR as a signal of T and T-derived traits. More research is needed to test the relationship between pubertal T and fWHR. In particular, studies should target diverse environmental settings given that T is responsive to socio-ecological inputs (e.g., Ellison et al., 2002). We are currently examining sex differences in fWHR during puberty in two datasets, which we hope will further clarify the T-fWHR relationship.

## Author contributions

Analysis and interpretation of data: CH-S, GR; Drafting of manuscript: CH-S, GR; Revising the manuscript critically for important intellectual content: CH-S, GR, TS, MG, SG; Approval of the version of the manuscript to be published: CH-S, GR, KS, TS, MG, SG.

### Conflict of interest statement

The authors declare that the research was conducted in the absence of any commercial or financial relationships that could be construed as a potential conflict of interest.
