# Corrigendum: Response: Commentary: Facial Width-to-Height Ratio (fWHR) Is Not Associated With Adolescent Testosterone Levels

**DOI:** 10.3389/fpsyg.2018.00541

**Published:** 2018-04-16

**Authors:** Psychology Editorial Office

**Affiliations:** Frontiers Media SA, Lausanne, Switzerland

**Keywords:** facial width-to-height ratio, testosterone, puberty, adolescence, sex hormones, facial dimorphism, secondary sexual characteristics

An incorrect version of this article was accepted for publication and published. This corrigendum has been submitted to correct this error. In addition, the author list has been updated to include George B. Richardson, who was missing in the incorrect version.

The original article has been updated and the previous incorrect version is available as supplementary material.

## Conflict of interest statement

The author declares that the research was conducted in the absence of any commercial or financial relationships that could be construed as a potential conflict of interest.

