# Supplementary material for: Response: Commentary: Facial Width-to-Height Ratio (fWHR) Is Not Associated with Adolescent Testosterone Levels
Source: Front Psychol. 2018 Feb 19;9:160. doi: 10.3389/fpsyg.2018.00160 (PMC5827542; doi:10.3389/fpsyg.2018.00160)
Supplement: Supplementary file 1 [file DataSheet1.pdf]

frontiers in Psychology

GENERAL COMMENTARY
published: 19 February 2018
doi: 10.3389/fpsyg.2018.00160

Response: Commentary: Facial Width-to-Height Ratio (fWHR) Is Not Associated with Adolescent Testosterone Levels

Carolyn R. Hodges-Simeon[1], Katherine N. H. Sobraske[2], Theodore Samore[3], Michael Gurven[2] and Steven J. C. Gaulin[2]*

[1] *Department of Anthropology, Boston University, Boston, MA, United States,* [2] *Department of Anthropology, University of California, Santa Barbara, Santa Barbara, CA, United States,* [3] *Department of Anthropology, University of California, Los Angeles, Los Angeles, CA, United States*

Keywords: facial width-to-height ratio, testosterone, puberty, adolescence, sex hormones, facial dimorphism, secondary sexual characteristics

A commentary on

Commentary: Facial Width-to-Height Ratio (fWHR) Is Not Associated with Adolescent Testosterone Levels
by Welker, K. W., Bird, B. M., and Arnocky, S. (2016). Front. Psychol. 7:1745. doi: 10.3389/fpsyg.2016.01745

OPEN ACCESS

Edited by:
Danielle Sulikowski, Charles Sturt University, Australia

Reviewed by:
Barnaby James Wyld Dixson, The University of Queensland, Australia

****Correspondence:***
Carolyn R. Hodges-Simeon
crhodges@bu.edu

Specialty section:
This article was submitted to Evolutionary Psychology, a section of the journal Frontiers in Psychology

Received: *09 August 2017*
Accepted: *31 January 2018*
Published: *19 February 2018*

Citation:
Hodges-Simeon CR, Sobraske KNH, Samore T, Gurven M and Gaulin SJC (2018) Response: Commentary: Facial Width-to-Height Ratio (fWHR) Is Not Associated with Adolescent Testosterone Levels. Front. Psychol. 9:160. doi: 10.3389/fpsyg.2018.00160

We thank Welker et al. (2016) for their interesting commentary and helpful additional analysis of our recent article. Given continuing interest in the relationships between facial width-to-height ratio (fWHR) and behaviors such as aggression and dominance (e.g., Carré and McCormick, 2008; Ozener, 2012; Lefevre et al., 2014; Geniole et al., 2015), it is important to address the relationship between pubertal testosterone (T) and facial development.

Welker et al. (2016) contend that Hodges-Simeon et al. (2016) use an overly liberal age range in their study design. The sample in question ranges from 8 to 22—a period of enormous phenotypic change in males. Any secondary sexual characteristic—i.e., those traits under the ultimate influence of sexual selection and often the proximate influence of androgens like T—shows strong, observable changes during this time. Therefore, the observation that both T and T-dependent traits are more masculinized in a 20-year-old male compared to his 10-year-old self is a banal fact. Barring a rare pathology, a 20-year-old will have a substantially lower voice, taller height, and greater T than he did when he was 10. Because of this, any cross-sectional dataset that includes 10-to-20 year olds will show a strong association between age and any T-dependent secondary sexual characteristics. Therefore any purported secondary sexual characteristics must pass an initial, "easy" test: a significant zero-order correlation with age (and, arguably, T) in a sample with this kind of age range. For example, vocal fundamental frequency (i.e., pitch) strongly correlates with both age ($r = -0.78$, $p < 0.001$) and T ($r = -0.75$, $p < 0.001$) in this sample (Hodges-Simeon et al., 2015). Height shows a slightly stronger association with both age ($r = -0.85$, $p < 0.001$) and T ($r = -0.78$, $p < 0.001$). Critically, fWHR could not pass this initial, low bar; we found no relationship between fWHR and age nor between fWHR and T. This finding alone casts substantial doubt on the possibility that fWHR is influenced by pubertal T. This is in stark comparison to the strong age- and T-associated changes in the three other masculinity ratios (all utilizing dimensions of the lower face and jaw) measured by Hodges-Simeon et al. (2016). Indeed—as Welker et al. (2016) point out—most phenotypic change usually happens between the ages of 12–16 in samples from energy-abundant countries. As the authors concede, an age range of 12–18 would be more appropriate for the Tsimane (where development is slower than in the US and growth is stunted), yet even an expanded age range did not yield significant results. While we agree that a narrower age

range may have improved the study in other ways and that the titular use of "adolescent" may create slightly different expectations, the liberal age range (and larger N) used should make the hypothesized effect of T on fWHR easier to detect rather than more difficult. For instance, the correlation between age and T is stronger for the entire sample ($r = 0.83$, $p < 0.001$) than for the limited, 12-to-16 sample ($r = 0.57$, $p < 0.001$).

Hodges-Simeon et al. (2016) control for age as a second pass in order to provide stronger, more convincing evidence for the influence of T on facial morphology. For instance, vocal fundamental frequency retains an association with T when the age is controlled ($r = -0.38$, $p < 0.01$; Hodges-Simeon et al., 2015). It is certainly true that fWHR and T were correlated when age was controlled, despite failing the initial test. We were confused by this relationship and address it in the discussion, pointing to a potential residual effect of prenatal T as a possible explanation (however, see Whitehouse et al., 2015).

Further, the literature provides a proximate understanding of the pathway through which T affects fundamental frequency via androgen receptors on the vocal folds (Voelter et al., 2008). We have no similar leads on how T might affect fWHR. In addition, adult fWHR is not associated with either prenatal (Whitehouse et al., 2015) or adult testosterone (Bird et al., 2016). Finally, it's not clear that fWHR is sexually dimorphic (Kramer et al., 2012; Lefevre et al., 2012; Ozener, 2012; Kramer, 2017; however, see Weston et al., 2007; Geniole et al., 2015); this unstable relationship with sex is problematic for the claim that fWHR is influenced by pubertal T.

Welker et al. (2016) contend that T was only transformed for the multiple regression model, but not the zero-order correlations. This was not the case. We see that the authors might have been confused by the description of our data analysis (i.e., "For regression analyses, T, height, strength, voice pitch, and age were log-transformed to match Pearson's correlation assumption of normality."); however, the same transformed variables were used to generate both zero-order and multivariate correlations.

CONCLUSION

We appreciate Welker et al.'s (2016) attention to our study and the topic of potential hormonal influences of adolescent facial growth. The research on fWHR thus far has been intriguing—in particular, findings on the associations between low fWHR and rated or actual aggression, dominance, strength (e.g., Carré and McCormick, 2008; Ozener, 2012; Lefevre et al., 2014; Geniole et al., 2015). Given these findings, we began this study with an expectation of finding a relationship between T and fWHR; however, we stand by our initial claim that a *robust* relationship is simply not there. The special circumstance in which fWHR is related to T in this dataset (i.e., only when age is controlled and only between the ages of 12–16 in a bivariate sense) is indeed confusing and deserves additional attention. However, it is important to look beyond the p-values to the broader theoretical and empirical context of a particular result. In a word, the results have to *make sense*.

Nevertheless, ours is a single study; future research is needed to confidently rule out the relationship between pubertal T and fWHR. In particular, these studies should target diverse environmental settings as T is responsive to socioecological inputs (e.g., Ellison et al., 2002). We are currently examining sex differences in fWHR during puberty in two datasets, which we hope will further clarify the T-fWHR relationship. Very few studies on the social perception of fWHR have used experimental manipulations of fWHR (however see Hehman et al., 2015). Because faces comprise a complex suite of intercorrelated traits, and humans are highly sensitive to even very small variations in facial dimensions, any study of fWHR must be mindful of whether and how much this singular dimension co-occurs with other aspects of craniofacial masculinity (Dixson, 2017). Future research on fWHR should identify (1) whether certain components of fWHR (e.g., nose length vs. philtrum length vs. eyebrow height) contributes more to social perceptions, and (2) the degree to which fWHR correlates with other sexually dimorphic, T-dependent facial traits (e.g., mandibular length and breadth). Multilevel selection experiments could be useful for this goal (see Brooks et al., 2015).

AUTHOR CONTRIBUTIONS

Analysis and interpretation of data: CH-S; Drafting of manuscript: CH-S; Revising the manuscript critically for important intellectual content: CH-S, TS, MG, SG; Approval of the version of the manuscript to be published: CH-S, KS, TS, MG, SG.

REFERENCES

Bird, B. M., Jofré, V. S. C., Geniole, S. N., Welker, K. M., Zilioli, S., Maestripieri, D., et al. (2016). Does the facial width-to-height ratio map onto variability in men's testosterone concentrations? *Evol. Hum. Behav.* 37, 392–398. doi: 10.1016/j.evolhumbehav.2016.03.004

Brooks, R. C., Shelly, J. P., Jordan, L. A., and Dixson, B. J. (2015). The multivariate evolution of female body shape in an artificial digital ecosystem. *Evol. Hum. Behav.* 36, 351–358. doi: 10.1016/j.evolhumbehav.2015.02.001

Carré, J. M., and McCormick, C. M. (2008). In your face: facial metrics predict aggressive behaviour in the laboratory and in varsity and professional hockey players. *Proc. Biol. Sci.* 275, 2651–2656. doi: 10.1098/rspb.2008.0873

Dixson, B. J. W. (2017). "Facial width-to-height ratio and dominance," in *Encyclopedia of Evolutionary Psychological Science*, eds T. K. Shackelford and V. A. Weekes-Shackelford (Cham: Springer).

Ellison, P. T., Bribiescas, R. G., Bentley, G. R., Campbell, B. C., Lipson, S. F., Panter-Brick, C., et al. (2002). Population variation in age-related decline in male salivary testosterone. *Hum. Reprod.* 17, 3251–3253. doi: 10.1093/humrep/17.12.3251

Geniole, S. N., Denson, T. F., Dixson, B. J., Carré, J. M., and McCormick, C. M. (2015). Evidence from meta-analyses of the facial width-to-height ratio as an evolved cue of threat. *PLoS ONE* 10:e0132726. doi: 10.1371/journal.pone.0132726

Hehman, E., Leitner, J. B., Deegan, M. P., and Gaertner, S. L. (2015). Picking teams: when dominant facial structure is preferred. *J. Exp. Soc. Psychol.* 59, 51–59. doi: 10.1016/j.jesp.2015.03.007

Hodges-Simeon, C. R., Gurven, M., and Gaulin, J. C. (2015). The low male voice is a costly signal of phenotypic quality among Bolivian adolescents. *Evol. Hum. Behav.* 36, 294–302. doi: 10.1016/j.evolhumbehav.2015.01.002

Hodges-Simeon, C. R., Hanson-Sobraske, K., Samore, T., Gurven, M., and Gaulin, S. J. C. (2016). fWHR is not associated with pubertal testosterone. *PLoS ONE* 4:e0153083. doi: 10.1371/journal.pone.0153083

Kramer, R. S. S. (2017). Sexual dimorphism of facial width-to-height ratio in human skulls and faces: a meta-analytical approach. *Evol. Hum. Behav.* 38, 414–420. doi: 10.1016/j.evolhumbehav.2016.12.002

Kramer, R. S., Jones, A. L., and Ward, R. (2012). A lack of sexual dimorphism in width-to-height ratio in White European faces using 2D photographs, 3D scans, and anthropometry. *PLoS ONE* 7:e42705. doi: 10.1371/journal.pone.0042705

Lefevre, C. E., Lewis, G. J., Bates, T. C., Dzhelyova, M., Coetzee, V., Deary, I. J., et al. (2012). No evidence for sexual dimorphism of facial width-to-height ratio in four large adult samples. *Evol. Hum. Behav.* 33, 623–627. doi: 10.1016/j.evolhumbehav.2012.03.002

Lefevre, C. E., Wilson, V. A., Morton, F. B., Brosnan, S. F., Paukner, A., and Bates, T. C. (2014). Facial width-to-height ratio relates to alpha status and assertive personality in capuchin monkeys. *PLoS ONE* 9:e93369. doi: 10.1371/journal.pone.0093369

Ozener, B. (2012). Facial width-to-height ratio in a Turkish population is not sexually dimorphic and is unrelated to aggressive behavior. *Evol. Hum. Behav.* 33, 169–173. doi: 10.1016/j.evolhumbehav.2011.08.001

Voelter, C., Kleinsasser, N., Joa, I. P., Nowack, I., Martínez, R., Hagen, R., et al. (2008). Detection of hormone receptors in the human vocal fold. *Eur. Arch. Otorhinolaryngol.* 265, 1239–1244. doi: 10.1007/s00405-008-0632-x

Welker, K. M., Bird, B. M., and Arnocky, S. A. (2016). Commentary: facial width-to-height ratio (fWHR) is not associated with adolescent testosterone levels. *Front. Psychol.* 7:1745. doi: 10.3389/fpsyg.2016.01745

Weston, E. M., Friday, A. E., and Lio, P. (2007). Biometric evidence that sexual selection has shape the hominin face. *PLoS ONE* 2:e710. doi: 10.1371/journal.pone.0000710

Whitehouse, A. J., Gilani, S. Z., Shafait, F., Mian, A., Tan, D. W., Maybery, M. T., et al. (2015). Prenatal testosterone exposure is related to sexually dimorphic facial morphology in adulthood. *Proc. R. Soc. B* 282:20151351. doi: 10.1098/rspb.2015.1351

Conflict of Interest Statement: The authors declare that the research was conducted in the absence of any commercial or financial relationships that could be construed as a potential conflict of interest.

Copyright © 2018 Hodges-Simeon, Sobraske, Samore, Gurven and Gaulin. This is an open-access article distributed under the terms of the Creative Commons Attribution License (CC BY). The use, distribution or reproduction in other forums is permitted, provided the original author(s) and the copyright owner are credited and that the original publication in this journal is cited, in accordance with accepted academic practice. No use, distribution or reproduction is permitted which does not comply with these terms.
